# Endoscopic vacuum therapy and early surgical closure after pelvic anastomotic leak: meta-analysis of bowel continuity rates

**DOI:** 10.1093/bjs/znac158

**Published:** 2022-05-30

**Authors:** F Borja de Lacy, Kevin Talboom, Sapho X Roodbeen, Robin Blok, Anna Curell, Pieter J Tanis, Wilhelmus A Bemelman, Roel Hompes

**Affiliations:** Gastrointestinal Surgery Department, Hospital Clinic of Barcelona, University of Barcelona, Barcelona, Spain; Department of Surgery, Amsterdam University Medical Centres, University of Amsterdam, Cancer Centre Amsterdam, Amsterdam, the Netherlands; Department of Surgery, Amsterdam University Medical Centres, University of Amsterdam, Cancer Centre Amsterdam, Amsterdam, the Netherlands; Department of Surgery, Amsterdam University Medical Centres, University of Amsterdam, Cancer Centre Amsterdam, Amsterdam, the Netherlands; Gastrointestinal Surgery Department, Hospital Clinic of Barcelona, University of Barcelona, Barcelona, Spain; Department of Surgery, Amsterdam University Medical Centres, University of Amsterdam, Cancer Centre Amsterdam, Amsterdam, the Netherlands; Department of Oncological and Gastrointestinal Surgery, Erasmus MC, Rotterdam, the Netherlands; Department of Surgery, Amsterdam University Medical Centres, University of Amsterdam, Cancer Centre Amsterdam, Amsterdam, the Netherlands; Department of Surgery, Amsterdam University Medical Centres, University of Amsterdam, Cancer Centre Amsterdam, Amsterdam, the Netherlands

## Abstract

**Background:**

Endoscopic vacuum therapy (EVT) with or without early surgical closure (ESC) is considered an effective option in the management of pelvic anastomotic leakage. This meta-analysis evaluated the effectiveness of EVT in terms of stoma reversal rate and the added value of ESC.

**Methods:**

A systematic search of PubMed, MEDLINE, and the Cochrane Library was conducted in November 2021 to identify articles on EVT in adult patients with pelvic anastomotic leakage. The primary outcome was restored continuity rate. Following PRISMA guidelines, a meta-analysis was undertaken using a random-effects model.

**Results:**

Twenty-nine studies were included, accounting for 827 patients with leakage who underwent EVT. There was large heterogeneity between studies in design and reported outcomes, and a high risk of bias. The overall weighted mean restored continuity rate was 66.8 (95 per cent c.i. 58.8 to 73.9) per cent. In patients undergoing EVT with ESC, the calculated restored continuity rate was 82 per cent (95 per cent c.i. 50.1 to 95.4) as compared to 64.7 per cent (95 per cent c.i. 55.7 to 72.7) after EVT without ESC. The mean number of sponge exchanges was 4 (95 per cent c.i. 2.7 to 4.6) and 9.8 (95 per cent c.i. 7.3 to 12.3), respectively. Sensitivity analysis showed a restored continuity rate of 81 per cent (95 per cent c.i. 55.8 to 99.5) for benign disease, 69.0 per cent (95 per cent c.i. 57.3 to 78.7) for colorectal cancer, and 65 per cent (95 per cent c.i. 48.8 to 79.1) if neoadjuvant radiotherapy was given.

**Conclusion:**

EVT is associated with satisfactory stoma reversal rates that may be improved if it is combined with ESC.

## Teaser

Available literature suggests that endoscopic vacuum therapy is associated with a satisfactory stoma reversal rate, especially if combined with early surgical closure, but there is substantial heterogeneity and high risk of bias.

## Introduction

Anastomotic leakage is the most feared complication in colorectal surgery. This adverse event increases morbidity, mortality, and healthcare costs, and decreases health-related quality of life, and may increase the risk of locoregional recurrence^1–4^. Despite surgical advances and newly developed preventive strategies^5–10^, low anterior resection is still associated with anastomotic leak rates of about 10–15 per cent^1,11^.

A significant number of pelvic leaks do not heal or may develop into a chronic sinus^12,13^. This late complication has a substantial impact on quality of life, with symptoms such as pelvic pain, purulent discharge, or even septicaemia^14,15^. Borstlap and colleagues^16^ reported absence of long-term healing after 48 per cent of leaks^13^, and the stoma is never closed in half of all patients who develop an anastomotic leak. These data emphasize the need for more effective treatment strategies.

In 2008, a new treatment comprising endoscopic placement of a vacuum sponge into the abscess cavity was introduced, referred to as endoscopic vacuum therapy (EVT)^17^. The effectiveness of EVT has been explored in several cohort studies^18–20^, with increasing interest in this technique in most recent years. Early surgical closure (ESC) by transanal suturing of the defect after a few sponge exchanges may improve outcomes further, if technically feasible^21,22^. However, complete anastomotic healing might still be difficult to achieve, with a risk of recurrent sinus after an apparent healing.

The reported incidence of anastomotic healing after EVT varies from 56 to 100 per cent; this in part reflects lack of consensus on the definition of anastomotic healing^18,23^. Several studies have considered both complete and partial anastomotic healing as a primary outcome for therapeutic success owing to this heterogeneity^20^. A more objective endpoint that better reflects the success of therapy from a patient perspective is the rate of living with a functional anastomosis. Therefore, this systematic review and meta-analysis was designed to evaluate the effectiveness of EVT in treating patients with pelvic anastomotic leak based on stoma closure rate, and to assess whether the outcomes improve with ESC.

## Methods

### Study design and registration

This study was conducted in accordance with PRISMA guidelines^24^. The protocol was registered in PROSPERO, the International Prospective Register of Systematic Reviews (CRD42019118088).

### Search strategy and study selection

An expert librarian assisted with a systematic search conducted in PubMed, MEDLINE, and the Cochrane Library for relevant articles between inception and February 2019, with an update in November 2021. The search strategy and information resources are detailed in *Appendix S1*. RCTs and observational studies of patients with pelvic intestinal anastomotic leakage treated with EVT were included. Only manuscripts written in English, and for which the full text was available, were included. Case reports and case series with fewer than five patients were excluded, as were animal studies. If the same group published different articles in the same interval, only the largest study was included.

The literature search was performed independently by two authors in March 2019 and two authors in November 2021. Disagreements were settled by discussion between the two reviewers, and reasons for exclusion were recorded during the screening processes. References in relevant publications were searched manually for additional potentially eligible studies.

### Procedures and definitions

Treatment with EVT consisted of endoscopic placement of an open-pored polyurethane sponge into the abscess cavity. The procedure was performed as described in previous articles^17,21,25^. Sponges were replaced every 3–4 days, allowing continuous monitoring of the development of granulation tissue and preventing ingrowth of the sponge. The sponge was connected to a low-vacuum suction bottle to generate a negative pressure and continuous evacuation of pus. Although EVT without faecal diversion has been described, the anastomosis was generally defunctioned.

**Fig. 1:**
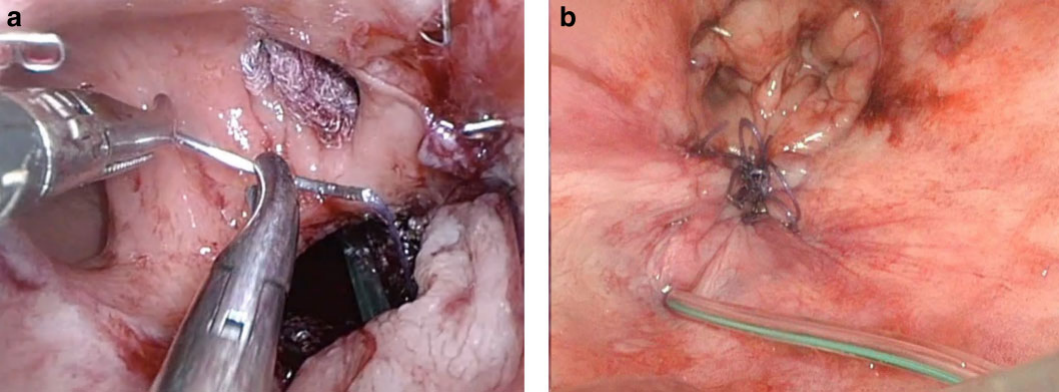
Early surgical closure **a** Anastomotic endoluminal view (closing of the anastomotic defect). **b** Final closure (reconstructed anastomosis with suction drain in the cavity).

ESC is a transanal surgical procedure, carried out under general anaesthesia, in which the anastomotic defect is closed. This can be considered when the abscess cavity is covered with granulation tissue and the rectal cuff can be reapproximated^21,22,26^. ESC is performed in the Lloyd-Davies position. Depending on the height of the anastomosis, an anal retractor (for example, Lonestar®; Cooper Surgical, Trumbull, CT, USA) or an endoscopic transanal platform, such as the flexible Gelpoint Path (Applied Medical, Rancho Santa Margarita, CA, USA), are used. A suction drain is placed in the cavity behind the reconstructed anastomosis, which results in obliteration of the cavity, after which the neorectum will stick to the sacrum (*Fig. 1*).

### Outcome measures and data collection

The primary outcome was restored gastrointestinal continuity at the end of follow-up. Secondary outcomes included time from index surgery to start of EVT, number of sponge exchanges, time to restored continuity, and short- and long-term complication rates.

The following data were extracted for each selected study: title, first author, year of publication, country, journal name, study design, strength of evidence, inclusion and exclusion criteria, sample size, patient characteristics (mean age, sex, BMI, neoadjuvant radiotherapy, ASA fitness grade, indication for index surgery), primary operative and postoperative outcomes (type of surgery, primary diverting stoma, time to diagnosis of anastomotic leakage), and EVT outcomes (technical details, time to initiation of EVT, number of sponge exchanges, need for secondary stoma, drain placement and removal, adjunct treatments, procedure-related events, and late complications).

### Quality assessment

Two authors independently assessed methodological quality using the Newcastle–Ottawa Scale (http://www.ohri.ca/programs/clinical_epidemiology/oxford.asp). A maximum of four points can be awarded for selection, two points for comparability, and three for outcome.

### Statistical analysis

Study and baseline characteristics are reported using descriptive statistics. A meta-analysis was performed for single proportions (restored continuity rate, and procedure-related and late complication rates) using a pooled random-effects analysis with inverse-variance weighting. The *I*^2^ value was calculated to assess statistical heterogeneity. A meta-analysis was undertaken for single means (interval from surgery to diagnosis of anastomotic leak, interval from surgery to start of EVT, number of sponge exchanges, and time to stoma reversal) from mean(s.d.) values reported in the studies. When data were missing, these were calculated from other data if possible (such as median or i.q.r.), using methods described by Wan and co-workers^27^. Both fixed-effect and random-effects analysis were performed using an inverse-variance method, and statistical heterogeneity was assessed by calculating the *I*^2^ value. Sensitivity analyses for restored continuity rates were conducted for EVT with or without ESC, benign disease (or more than 90 per cent benign disease among included patients) *versus* colorectal cancer (or over 90 per cent colorectal cancer among included patients), colorectal cancer with radiotherapy *versus* any type of disease without radiotherapy, and primary diverting stoma (or more than 80 per cent of included patients) *versus* no primary diverting stoma (or less 20 per cent of included patients). Publication bias was investigated by visual inspection of the funnel plot of restored continuity, and using the Peters’ test to assess linear regression of funnel plot asymmetry (based on sample size)^28^.

No comparative meta-analysis between EVT with or without ESC was undertaken because only single cohort studies were found; results are presented separately for the two subgroups. A meta-analysis of healed anastomosis rate was not done because of the high level of heterogeneity in definition of a healed anastomosis. Meta-analysis was performed using RStudio version 1.2.1335 (RStudio: Integrated Development for R; RStudio, PBC, Boston, MA, USA).

## Results

**Fig. 2:**
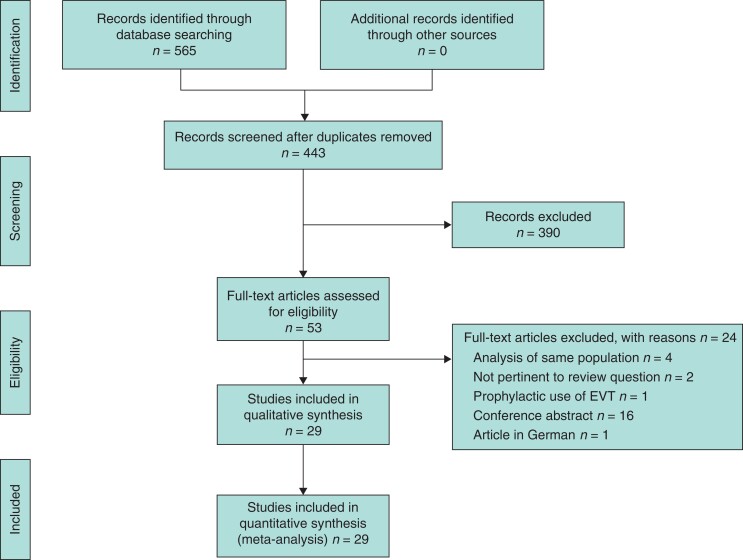
PRISMA flow diagram showing selection of articles for review EVT, endoscopic vacuum therapy.

The literature search yielded 442 records. After screening titles and abstracts, 53 articles were eligible for full-text review. Of these, 29 studies^18,19,21–23,25,29–51^ were finally included. Reasons for exclusion are shown in *Fig. 2*. No RCT was found. Six studies^22,29,31,45,47,50^ were cohort studies, including one that used matching to handle allocation bias. The remaining studies^18,19,21,23,25,30,32–44,46,48,49,51^ were case series from institutional databases. Four studies^21,22,25,45^ used ESC as an adjunct to EVT. However, the study by Huisman and colleagues^45^ was excluded from the subgroup analysis as it was not possible to extract specific information for the ESC cohort (3 patients, 15 per cent of the whole group).

Quality assessment of the included studies is reported in *Table S2*. The funnel plot appeared potentially asymmetrical, but Peters’ linear regression indicated no asymmetry in the funnel plot, indicating a low likelihood of publication bias (*P* = 0.356) (*Fig. S1*).

*Table 1* summarizes the characteristics of the included studies, accounting for a total of 827 patients. Surgery for colorectal cancer was the primary indication for surgery (613 of 817 patients, 75.0 per cent)^18,19,21–23,25,29–39,41–51^. Sixty-six patients (8 per cent) were treated for inflammatory bowel disease and 134 patients (16.4 per cent) had various underlying diseases as an indication for initial surgery^18,19,21–23,25,29–39,41–51^.

**Table 1:** Characteristics of included studies of endoscopic vacuum therapy for pelvic anastomotic leakage

Reference	Study design	Inclusion criteria	Indication	*n*	Male sex	Age (years)	Primary stoma	NART	Adjunct treatment (%)
**Mees *et al.*^29^**	Prospective matched cohort	Symptomatic leak after AR or IPAA	Rectal cancer and UC	5	4 of 5	47	n.a.	0 of 5	None
**Glitsch *et al.*^30^**	Prospective case series	Symptomatic leak after AR or colectomy with extraperitoneal anastomosis	Rectal cancer	17	14 of 17	61	8 of 17	9 of 17	15 Fibrin glue
**van Koperen *et al.*^18^**	Prospective case series	Symptomatic leak after AR or IPAA	Rectal cancer and UC	16	9 of 16	64	8 of 16	11 of 16	None
**von Bernstorff *et al.*^19^**	Prospective case series	Symptomatic leak after AR	Rectal cancer	26	21 of 26	58	14 of 26	14 of 26	None
**Chopra *et al.*^31^**	Retrospective cohort	Symptomatic leak after AR	Rectal cancer	5	n.a.	n.a.	n.a.	5 of 5	Fibrin glue: 2
**Riss *et al.*^32^**	Retrospective case series	Symptomatic leak after AR or Hartmann insufficiency	Rectal cancer	9	5 of 9	64	4 of 9	4 of 9	None
**Verlaan *et al.*^25^**	Prospective case series	Symptomatic leak after AR or IPAA	Rectal cancer and UC or FAP	6	5 of 6	50	0	1 of 6	ESC: 4 Clip: 1
**Srinivasamurthy *et al.*^33^**	Retrospective case series	Extraperitoneal anastomosis and symptomatic leak after AR or IPAA	Rectal cancer and UC	8	7 of 8	67	n.a.	7 of 8	None
**Nerup *et al.*^23^**	Retrospective case series	Symptomatic leak after AR	Rectal cancer	13	11 of 13	64	13 of 13	6 of 13	None
**Keskin *et al.*^34^**	Retrospective case series	Symptomatic leak after AR, IPAA or IRA	Rectal cancer, FAP and diverticular disease	15	7 of 15	55	14 of 15	6 of 15	None
**Arezzo *et al.*^35^**	Retrospective case series	Symptomatic leak after AR, TEM or STARR	Rectal cancer, rectal adenoma, RV fistula	14	7 of 14	68	8 of 14	7 of 14	Glue and clip
**Strangio *et al.*^36^**	Prospective case series	Symptomatic leak after AR, IPAA or left colectomy	Rectal cancer, endometriotic nodule, UC, colonic cancer, diverticulitis	25	18 of 25	67	13 of 25	8 of 25	None
**Kuehn *et al.*^37^**	Retrospective case series	Symptomatic leak after AR, Hartmann insufficiency, IPAA, TEM or STARR	Rectal cancer, diverticulitis, UC, rectal perforation, UC, fistula	41	31 of 41	70	19 of 19	12 of 41	None
**Mussetto *et al.*^38^**	Retrospective case series	Symptomatic leak after AR	Rectal cancer	11	6 of 11	71	n.a.	5 of 11	None
**Milito *et al.*^39^**	Prospective case series	AL of low rectal anastomosis	Rectal cancer	14	10 of 14	65	14 of 14	14 of 14	None
**Mencio *et al.*^40^**	Retrospective case series	Patients with different GI leaks	n.a.	10	5 of 10	55	7 of 10	n.a.	None
**Jimenez-Rodriguez *et al.*^41^**	Prospective case series	Symptomatic leak after AR or Hartmann insufficiency	Rectal cancer	22	18 of 22	65	13 of 22	17 of 22	Fibrin glue: 10
**Borstlap *et al.*^21^**	Prospective case series	Symptomatic leak after AR	Rectal cancer	30	19 of 30	66	23 of 30	22 of 30	ESC: 30
**Rottoli *et al.*^42^**	Prospective case series	Symptomatic leak after IPAA	UC and FAP	8	n.a.	37	8 of 8	0 of 8	None
**Katz *et al.*^43^**	Retrospective case series	Symptomatic leak after AR, IPAA	Rectal cancer, Hirschprung, FAP, ovarian cancer with rectal involvement	6	5 of 6	54	3 of 6	n.a.	None
**Wasmann *et al.*^22^**	Retrospective cohort	Symptomatic leak after IPAA	UC	18	12 of 18	41	1 of 18	0 of 18	ESC: 18
**Boschetti *et al.*^44^**	Retrospective case series	Symptomatic leakage	Colonic cancer, rectal cancer, sigmoiditis	29	22 of 29	68	12 of 29 (41.4)	19 of 29 (65.5)	None
**Huisman *et al.*^45^**	Retrospective cohort	Symptomatic leakage after rectal surgery	Rectal cancer, IBD	20	14 of 20	64	14 of 20	14 of 20	ESC: 3
**Kantowski^46^**	Retrospective case series	AL after colorectal resection	Rectal cancer, diverticular disease, IBD, ischaemia	89	68 of 89	58	87 of 89	27 of 89	Transanal rinsing therapy after EVT: 58
**Abdalla *et al.*^47^**	Prospective case series	Leakage after elective proctectomy	Rectal cancer, IBD	47	36 of 47	65	40 of 47	27 of 47	None
**Weréen *et al.*^48^**	Retrospective cohort study	Symptomatic leakage after AR	Rectal cancer	14	9 of 14	64	12 of 14	13 of 14	None
**Kühn *et al.*^49^**	Prospective case series	Colorectal defects	Rectal cancer, IBD, diverticular disease, other malignancies, perforation	281	186 of 281	65	224 of 281	95 of 281	None
**Jagielski *et al.*^50^**	Prospective cohort study	AL after rectal cancer surgery	Rectal cancer	18	18 of 18	61	8 of 18	16 of 18	None
**Keshvari *et al.*^51^**	Prospective case series	AL after LAR	Rectal cancer	10	6 of 10	56	10 of 10	10 of 10	None

Values in parentheses are percentages. NART, neoadjuvant radiotherapy; (L)AR, (low) anterior resection; IPAA, ileal pouch–anal anastomosis; UC, ulcerative colitis; n.a., not available; FAP, familial adenomatous polyposis; ESC, early surgical closure; IRA, ileorectal anastomosis; TEM, transanal endoscopic microsurgery; STARR, stapled transanal rectal resection; RV, rectovaginal; GI, gastrointestinal; IBD, inflammatory bowel disease; AL, anastomotic leakage; EVT, endoscopic vacuum therapy.

### Baseline characteristics

The pooled mean age for all patients was 62.9 years, and the overall male to female ratio, calculated on the basis of the studies reporting sex, was 2.5 : 1. Weighted mean BMI was 25.4 kg/m^2^ (*Table 2*). The weighted mean time interval between index surgery and diagnosis of leakage was 20.2 (95 per cent c.i. 15.9 to 24.6) days.

**Table 2:** Baseline characteristics of included studies

	No. of studies	Total		No ESC		ESC	
		*n*	Pooled value (%)*	*n*	Pooled value (%)*	*n*	Pooled value (%)*
**Patient characteristics**							
Men	27	573 of 814	70.4	537 of 760	70.7	36 of 54	67
Age (years)	27	804	62.9†	750	63.4†	54	56†
BMI (kg/m^2^)	10	197	25.4†	149	25.5†	48	25†
Neoadjuvant radiotherapy	27	369 of 811	45.5	346 of 757	45.7	23 of 54	43
**Indication for primary surgery**							
Colorectal cancer	28	613 of 817	75.0	582 of 763	76.3	31 of 54	57
IBD	28	66 of 817	8.1	43 of 763	5	23 of 54	43
Other	28	134 of 817	16.4	134 of 763	17.6	0 of 54	0
**Primary stoma (created during index surgery)**	24	577 of 776	74.4	553 of 722	73.6	24 of 54	44
**Secondary stoma (created after index surgery)**	23	119 of 687	17.3	86 of 613	14.0	30 of 54	56
**EVT in outpatient setting**	9	216 of 423	51.1	216 of 423	51.1	0	0
**Duration of follow-up (months)**	13	246	19.4†	170	17.5†	54	30†

*Unless indicated otherwise; †mean value. ESC, early surgical closure; *n*, number of patients; IBD, inflammatory bowel disease; EVT, endoscopic vacuum therapy.

Of 776 patients, 577 (74.4 per cent) had a diverting stoma after primary surgery, and 119 of 687 (17.3 per cent) received a secondary stoma following anastomotic leakage after the primary resection (*Table 2*). The pooled mean follow-up for all patients was 19.4 months. Among patients undergoing EVT without ESC, 553 of 722 (73.6 per cent) had faecal diversion with a primary stoma, 86 of 613 (14.0 per cent) had a secondary stoma, and mean follow-up was 17.5 months. In patients undergoing EVT with ESC, 24 of 54 patients had faecal diversion (44 per cent) with primary stoma, 30 of 54 had faecal diversion with a secondary stoma (55 per cent) and the mean follow-up was 29.8 months.

### Outcomes of endoscopic vacuum therapy

**Fig. 3:**
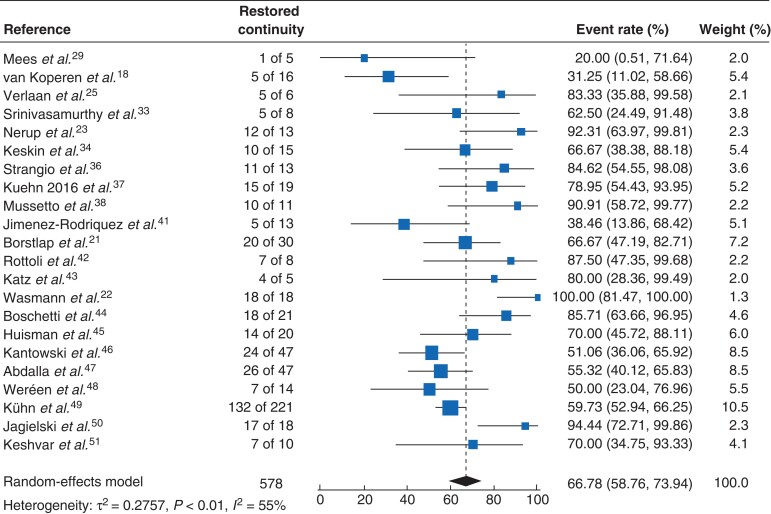
Forest plot showing restored continuity rates after endoscopic vacuum therapy Event rates are shown with 95 per cent confidence intervals.

*Table 3* shows the general outcomes of EVT, including all studies independent of adjunct ESC. Random-effects meta-analysis showed that the weighted mean rate of restored continuity after stoma formation (either primary or secondary) was 66.8 (95 per cent c.i. 58.8 to 73.9) per cent (*I*^2^ = 55 per cent) (*Fig. 3*)^18,21–23,25,29,33,34,36–38,41–51^. The calculated mean rate of procedure-related complications was 6.7 (4.7 to 9.6) per cent^18,19,21–23,29–39,41–47,50,51^. Healed anastomosis rates and definitions are presented separately for the included studies in *Table S1*. From the available information, EVT could be continued in an outpatient setting in 216 patients (representing 51.1 per cent of the total of 423 patients from studies reporting this information)^19,29,30,34,35,41,44,48,49^. The documented late complication rate was 10.8 (6.8 to 16.7) per cent among 21 studies comprising 440 patients^18,19,21–23,29,30,32–39,41,42,44,45,47,51^.

**Table 3:** Pooled outcomes after endoscopic vacuum therapy in patients with pelvic anastomotic leakage

	Total			No ESC			ESC		
	No. of studies	*n*	Pooled value (%)*	No. of studies	*n*	Pooled value (%)*	No. of studies	*n*	Pooled value (%)*
**Interval from surgery to AL diagnosis (days)**	16	272	20.2 (15.9, 24.6)†	12	198	23.5 (17.2, 29.9)†	3	54	15 (8.3, 22.5)†
**Interval from surgery to EVT (days)**	15	265	35.9 (27.8, 44.0)†	11	191	38.3 (28.8, 47.8)†	3	54	23 (9.1, 37.0)†
**No. of sponges used**	26	710	9.1 (7.0, 11.3)†	22	636	9.8 (7.3, 12.3)†	3	54	4 (2.7, 4.6)†
**Anastomotic function**									
Restored continuity (%)	22	578	66.8 (58.8, 73.9)	18	505	64.7 (55.7, 72.7)	3	54	82.0 (50.1, 95.4)
Time to restored continuity (months)‡	7	114	5.1 (3.3, 6.9)†	3	51	4 (2.5, 4.9)†	3	43	2 (0.9, 4.0)†
**Complications**									
Procedure-related	25	516	6.7 (4.7, 9.6)	22	461	10.2 (6.7, 15.1)	2	48	2 (0, 0.1)
Late (during follow-up)	21	440	10.8 (6.8, 16.7)	18	372	9.7 (6.0, 15.3)	2	48	14 (1.0, 72.3)

Values in parentheses are 95 per cent confidence intervals. *Unless indicated otherwise; †mean. ‡After diagnosis of anastomotic leakage (AL). ESC, early surgical closure; *n*, number of patients; EVT, endoscopic vacuum therapy.

### Time to start of endoscopic vacuum therapy

Several authors have suggested that the timing of EVT may influence treatment outcomes. However, these analyses usually focused on anastomotic healing, and only three reported data on stoma reversal rate at the end of follow-up. Borstlap and colleagues^21^ found that starting EVT within the first 21 days was associated with a non-significant increase in stoma reversal rate (73 *versus* 60 per cent; median follow-up 14 months). With a median follow-up of 10 months, Huisman *et al*.^45^ reported a cumulative probability of stoma removal of 77 (95 per cent c.i. 22 to 93) per cent when EVT was started within the first 21 days, compared with 70 (23 to 88) per cent in the late-initiation group (*P* = 0.31). Abdalla and co-workers^47^ documented a higher stoma reversal rate when EVT was started 15 days after diagnosis of anastomotic leakage than when it was initiated later (72.4 *versus* 27.8 per cent; *P* = 0.003).

### Endoscopic vacuum therapy with or without early surgical closure

**Fig. 4:**
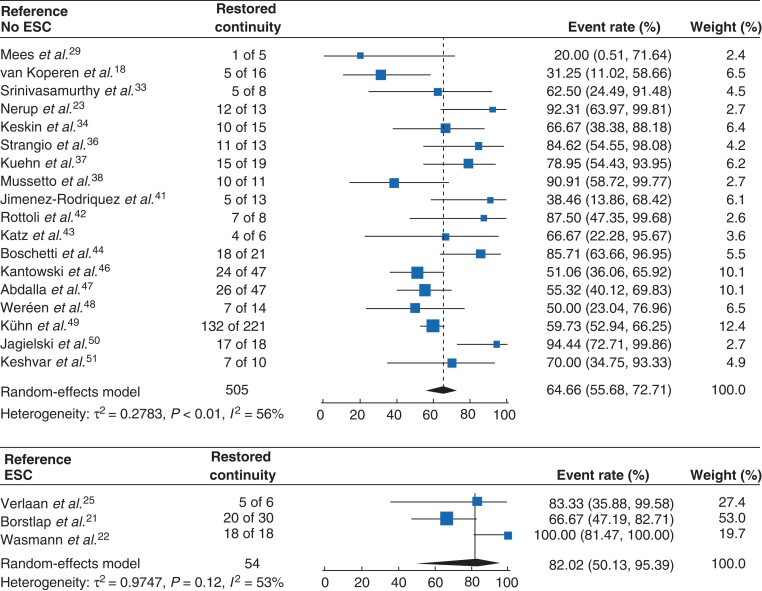
Forest plot showing restored continuity rates after endoscopic vacuum therapy with or without early surgical closure Event rates are shown with 95 per cent confidence intervals. ESC, early surgical closure.

Fifty-four patients had EVT with ESC, of whom 23 underwent ileal pouch–anal anastomosis (IPAA). Regarding baseline characteristics, primary resection for colorectal cancer was performed in 31 of 54 patients who underwent EVT with ESC (57 per cent) and in 582 of 763 (76.3 per cent) without ESC. Corresponding proportions neoadjuvant radiotherapy were 23 of 54 (43 per cent) and 346 of 757 (45.7 per cent), respectively. Random-effects meta-analysis showed that the weighted mean rate of restoration of continuity in the ESC group was 82 per cent (95 per cent c.i. 50.1 to 95.4)^21,22,25^, which was 64.7 per cent (95 per cent c.i. 55.7 to 72.7) in the group without ESC (*Table 3* and *Fig. 4*). The mean number of sponge exchanges was 4 (95 per cent c.i. 2.7 to 4.6) in the EVT with ESC group, compared to a mean of 9.8 (95 per cent c.i. 7.3 to 12.3) in the EVT-only group.

### Sensitivity analysis

Sensitivity analysis showed a restored continuity rate of 81.0 (95 per cent c.i. 55.8 to 99.5) per cent for patients with benign disease, 69.0 (57.3 to 78.7) per cent for those with colorectal cancer, and 65.5 (48.8 to 79.1) per cent if neoadjuvant radiotherapy was administered (*Table 4*). The restored continuity rate was 61.9 (53.4 to 69.7) per cent in patients who received a primary diverting stoma, and 83.1 (66.2 to 92.5) per cent among those without a primary stoma.

**Table 4:** Sensitivity analysis for restored continuity in different subgroups of patients undergoing endoscopic vacuum therapy for pelvic anastomotic leakage

	No. of studies	*n*	Restored continuity rate (%)
**Benign disease (or > 90%)**	5	39	81.0 (55.8, 99.5)
**Colorectal cancer (or ≥ 90%)**	11	201	69.0 (57.3, 78.7)
**Colorectal cancer with radiotherapy**	5	76	65.5 (48.8, 79.1)
**Any type of disease, no radiotherapy**	6	57	70 (38.8, 89.7)
**Primary diverting stoma (or ≥ 80%)**	11	420	61.9 (53.4, 69.7)
**No primary stoma (or ≤ 20%)**	3	81	83 (66.2, 92.5)

Values in parentheses are 95 per cent confidence intervals. *n*, number of patients.

## Discussion

In this systematic review including 29 studies, EVT was associated with successful restoration of continuity, with a functional anastomosis in two-thirds of patients. The stoma reversal rate at the end of follow-up seemed to be higher for patients treated with combined EVT plus ESC compared with EVT alone. Most studies were retrospective cohort studies, with a large difference in cohort size ranging from 5 to 281 patients, and a wide variety of underlying diseases as well as primary treatment modalities (colonic anastomosis or IPAA, with or without neoadjuvant radiotherapy). This resulted in a high risk of bias. Therefore, the present findings should be interpreted carefully for the different subgroups and indications. Nevertheless, these results justify further investigation in larger prospective series and international registries with extended follow-up, given the ethical and other practical and methodological issues related to controlled randomized conditions in this specific population.

EVT aims to control pelvic sepsis and gradually reduce the size of the sinus. In the original publication, Weidenhagen and colleagues^17^ reported definitive anastomotic healing in more than 96 per cent of patients. Since then, a number of observational studies^17–19,23,29–31,35–39,41^ have been published, with variable success rates in heterogeneous patient populations. Meta-analyses^20,52–54^ have been undertaken in this area. The present review is an update, with a substantially larger number of studies and patients, which also enabled sensitivity analyses of clinically relevant subgroups. Furthermore, the additional value of ESC was not analysed in the previous reviews.

There is a lack of consensus on how to classify anastomotic healing after leakage. Across the included studies, there was a wide range of definitions. Imaging and/or endoscopic confirmation was included in some of these, whereas others did not describe any specific criteria at all. This hinders the ability to compare results and, more importantly, underlines the need for consensus on an objective and reproducible universal definition. For future research, objective measures for anastomotic healing should be used, such as the absence of any extraluminal air or fluid on CT with rectal contrast, and absence of symptoms indicative of reactivation of leakage following stoma closure.

Among the currently used definitions, a healed anastomosis may refer to true healing but also pelvic symptom containment. However, restored continuity (without the need for any major salvage surgery) is a hard endpoint that reflects the rate of functional anastomoses. Several studies have reported permanent stoma rates after conventional management of anastomotic leakage. Maggiori and co-workers^55^, with a median follow-up of 3 years, reported a 36 per cent rate in patients with symptomatic anastomotic leak treated with a secondary stoma. In the 2011 Dutch Surgical Colorectal Audit, Borstlap *et al*.^13^ analysed 998 patients who underwent low anterior resection, and reported an early anastomotic leak rate of 13.4 per cent. The rate of unintentional permanent stoma after anastomotic leak was 46 per cent after a median of 43 months, which is similar to the 51 per cent rate in the Dutch TME trial^16^ with 7 years of follow-up. The findings of the present meta-analysis showed that, with a median follow-up of less than 2 years, EVT was associated with a long-term stoma rate of 33 per cent, which is somewhere between the permanent stoma rates ranging from 24 to 49 per cent in previously published meta-analyses^20,52,53^. This 33 per cent stoma rate seems acceptable, but at the same time does not convincingly show better stoma-free survival than that achieved with conventional leakage management. This might represent selection bias, with more severe leaks treated using EVT, and more asymptomatic radiological leaks managed in a conventional passive way.

The addition of ESC was associated with better outcomes, with a long-term stoma rate of 18 per cent. However, it should be noted that the proportion of IPAAs was relatively high in the ESC group compared with that among patients who received EVT alone, and these results cannot be extrapolated to rectal cancer populations undergoing neoadjuvant radiotherapy. Anastomotic leakage severity scores need to be developed for the purpose of better comparison between treatment strategies^56^.

Establishing the cost-effectiveness of a new therapy is important before its use becomes widespread in reimbursed healthcare systems. The financial impact of treating a patient with anastomotic leakage is already high, with additional costs of approximately €18 000 compared with those for patients with no leak^57^. It has been reported previously that five patients must be treated with EVT and ESC in order to save one extra anastomosis, compared with standard passive anastomotic leak management^21^. The present study found that EVT with ESC required six fewer endoscopies for sponge replacement than EVT alone. This implies a direct reduction in resources, but also in time to completion of treatment. Moreover, the suggested improved clinical outcomes observed with the addition of ESC indicate potential cost-effectiveness, but this has to be confirmed in properly designed studies.

The development of a pelvic anastomotic leak may lead to significant postoperative bowel dysfunction. For this reason, in addition to studying how these leaks are treated using hard endpoints such as stoma closure, it is important to include functional and quality-of-life outcomes. The ability to control pelvic sepsis and close a defect earlier by means of EVT and ESC, with fewer sponge replacements, may also improve function. This was shown recently in a cohort study^22^ of patients undergoing IPAA, which found that EVT with ESC was associated with preservation of pouch function and preclusion of pouch failure, in contrast to conventional leak management. Unfortunately, very few studies have reported on function after EVT with or without ESC; this represents an important knowledge gap that should also be addressed in future studies.

Of all the factors that may increase the effectiveness of EVT, it seems that early diagnosis and initiation of treatment are crucial^52^. Late initiation of EVT might be ineffective owing to the retraction of the anastomotic edges and reduced pliability of the neorectum. An especially susceptibility group of patients are those with primary diversion and an asymptomatic anastomotic leak, in whom dehiscence may be diagnosed only after stoma reversal. Therefore, to detect occult leaks, and with the aim of initiating EVT as soon as possible, highly selective diversion with early C-reactive protein measurement in all patients receiving a pelvic anastomosis, followed by CT or endoscopy when necessary, is recommended^58^. The sensitivity analysis also hints in a similar direction, with a higher rate of restored continuity in patients without a primary stoma (83.1 *versus* 61.9 per cent).

This study has several limitations. The sample sizes of the included studies were mostly small and there was considerable heterogeneity among the inclusion criteria. Moreover, the studies had methodological limitations, mostly based on imperfect designs and reporting. The primary outcome—stoma reversal rate—was considered to be the rate at the end of the follow-up; nevertheless, additional stomas might have been created after manuscript publication, for example for a small persistent sinus or faecal incontinence. The majority of articles included patients with anastomotic leakage, but a few also included patients with rectal stump insufficiency following a low Hartmann’s procedure. These data could not be analysed separately and may be a source of bias.

## Supplementary Material

znac158_Supplementary_Data
