# Supplementary material for: Endoscopic vacuum therapy and early surgical closure after pelvic anastomotic leak: meta-analysis of bowel continuity rates
Source: Br J Surg. 2022 May 30;109(9):822–31. doi: 10.1093/bjs/znac158 (PMC10364759; doi:10.1093/bjs/znac158)
Supplement: znac158_Supplementary_Data [file znac158_supplementary_data.zip › Sup Table 1 - After minor revisions_.docx]

**Supplementary Table 1**: Definitions used for anastomotic healing/treatment success

| **Author** | **Definition healed/success** | **Follow-up modality** | **Healed anastomosis (%)** | **Days to healing** | **Follow-up** |
| --- | --- | --- | --- | --- | --- |
| van Koperen ^18^ | Definitive resolution of the sinus | Endoscopy | 9/16 (56%) | 40 (28-90)**^a^** | - |
| von Bernstorff ^19^ | Complete closure of the cavity | Endoscopy | 23/26 (88%) | 50 (10-221)**^a^** | - |
| Borstlap ^21^ | No signs of contrast extravasation during abdominal CT or contrast enema and an intact anastomosis during endoscopy | Endoscopy + CT with rectal contrast | 21/30 (70%) | 127 (14-722)^a^ | 14 months |
| Wasmann ^22^ | Anastomotic integrity was assessed endoscopically 2 weeks after surgical closure. subsequently, CT with intraluminal contrast was used to exclude presacral fluid collections. | Endoscopy + CT with intraluminal contrast | 18/18 (100%) | 30 (17-40)^b^ | 48 months |
| Nerup ^23^ | Closure of the perianastomotic abscess | Endoscopy | 13/13 (100%) | NR | - |
| Verlaan ^25^ | Closure of defect | Endoscopy + CT with enteral contrast | 6/6 (100%) | 13 (3-29)**^a^** | - |
| Mees ^29^ | Endoscopically proven closure of the insufficiency cavity with achievement  of the normal mucosa level. | Endoscopy | 5/5 (100%) | 45 (32-68)^a^ | - |
| Glitsch ^30^ | Complete closure of the cavity. | Endoscopy | 16/17 (94%) | 53 (10-221)^c^ | - |
| Chopra ^31^ | Complete healing of the anastomotic defect with intact mucosal covering | Endoscopy | NR | NR | - |
| Riss ^32^ | A cavity that is nearly closed and totally covered by granulation tissue. | Endoscopy | 6/9 (67%) | NR | - |
| Srinivasamurthy ^33^ | Complete closure or a reduction in the size of the abscess cavity | Endoscopy | 6/8 (75%) | NR | 41 months |
| Keskin ^34^ | None | Endoscopy | 12/15 (80%) | NR | - |
| Arezzo ^35^ | Complete restoration of the wall epithelium, confirmed by endoscopic examination with contrast injection during endoscopy | Endoscopy | 11/14 (89%) | 41 (8-114)**^a^** | - |
| Strangio ^36^ | Cavity less than 1 cm in diameter | Endoscopy | 22/25 (88%) | 28 (7-128)**^a^** | 9 months |
| Kuehn 2016 ^37^ | None | Endoscopy | 34/41 (83%) | NR | 36 months |
| Mussetto ^38^ | A decreased cavity covered with granulation tissue that did not allow the insertion of a new sponge. | Endoscopy, contrast barium enema | 10/11 (91%) | 37 (18-65)^c^ | 29 months |
| Milito ^39^ | Endoscopically proven closure of the insufficiency cavity with a normal mucosa. | Endoscopy | NR | 37 (19-55)**^a^** | - |
| Mencio ^40^ | Resolution of the leak or perforation with restoration  of GI continuity and the initiation of an oral diet. | Endoscopy | 6/10 (60%) | 23 (NR)^c^ | - |
| Jimenez-Rodriguez ^41^ | None | Endoscopy | 20/22 (91%) | 22 (15)^d^ | 12 months |
| Rottoli ^42^ | Closure of the defect after a progressive reduction in size of the cavity without signs of infection or complications, not requiring any other intervention than the follow-up pouchoscopy | Endoscopy | 8/8 (100%) | 60 (24-90)^b^ | 12 months |
| Katz ^43^ | None | CT with enema contrast + endoscopy | NR | NR | 28 months |
| Boschetti ^44^ | Cavity too small to introduce a new sponge | Endoscopy | 27/29 (93%) | 63 (NR)^a^ | 6 months |
| Huisman ^45^ | A cavity reduced in size and covered with granulation tissue that was too small to allow placement of a new endosponge | Endoscopy | 17/20 (85%) | 25 (3-115)^a^ | 10 months |
| Kantowski ^46^ | None | Endoscopy | 67/89 (75%) | NR | - |
| Abdalla ^47^ | No residual leak on the control contrast-enhanced enema | Contrast-enhanced enema | NR | NR | 35 months |
| Wereen ^48^ | *Not included as outcome* | NR | NR | NR | - |
| Kuhn 2020 ^49^ | Granulating closure of the cavity, more than 90% clean and granulating tissue, decreasing wound secretion, reduction of fibrinous tissue, and no interventional or surgical procedure required in the further course due to local wound healing and successful sepsis control (monitored clinically and by laboratory parameters). | Endoscopy | 256/281 (91%) | NR | - |
| Jagielski ^50^ | Resolution of clinical signs and complete resolution of an abscess with leak closure by granular tissue or as the resolution of clinical signs and reduction in the size of the abscess to a diameter below 30 mm (confirmed by imaging) with filling of an abscess with granulation tissue (confirmed by endoscopy), which allowed EVT to be completed. | Endoscopy + CT with intraluminal contrast | 17/18 (94%) | NR | 12 months |
| Keshvari ^51^ | Closure of the cavity as observed in the flexible rectoscopy examinations | Endoscopy | 8/10 (80%) | 118 (68)^d^ | - |

*GI: gastro-intestinal; CT: Computed Tomography; EVT: endoscopic vacuum therapy*

^a^ Median (range); ^b^ Median (IQR); ^c^ Mean (range); ^d^ Mean (SD)
