# Supplementary material for: Endoscopic vacuum therapy and early surgical closure after pelvic anastomotic leak: meta-analysis of bowel continuity rates
Source: Br J Surg. 2022 May 30;109(9):822–31. doi: 10.1093/bjs/znac158 (PMC10364759; doi:10.1093/bjs/znac158)
Supplement: znac158_Supplementary_Data [file znac158_supplementary_data.zip › Sup Table 2 - After minor revisions_.docx]

**Supplementary Table 2:** Quality Assessment through Newcastle Ottawa Scale

| **Author, year** | **Study design** | **Selection (NOS)** | **Comparability (NOS)** | **Outcome (NOS)** |
| --- | --- | --- | --- | --- |
| Van Koperen, 2009 ^18^ | Prospective | ⭑ ⭑ ⭑ | ⭑ ⭑ | ⭑ ⭑ |
| Von Bernstorff, 2009 ^19^ | Prospective | ⭑ ⭑ ⭑ | ⭑ ⭑ | ⭑ ⭑ |
| Borstlap, 2018 ^21^ | Prospective | ⭑ ⭑ ⭑ ⭑ | ⭑ ⭑ | ⭑ ⭑ ⭑ |
| Wasmann, 2018 ^22^ | Retrospective | ⭑ ⭑ ⭑ ⭑ | ⭑ ⭑ | ⭑ ⭑ |
| Nerup, 2013 ^23^ | Retrospective | ⭑ ⭑ ⭑ | ⭑ | ⭑ ⭑ |
| Verlaan, 2011 ^25^ | Prospective | ⭑ ⭑ ⭑ | ⭑ | ⭑ ⭑ ⭑ |
| Mees, 2008 ^29^ | Prospective | ⭑ ⭑ ⭑ | ⭑ | ⭑ ⭑ |
| Glitsch, 2008 ^30^ | Prospective | ⭑ ⭑ | ⭑ | ⭑ ⭑ |
| Chopra, 2009 ^31^ | Retrospective | ⭑ ⭑ | ⭑ | ⭑ ⭑ |
| Riss, 2010 ^32^ | Retrospective | ⭑ ⭑ | ⭑ | ⭑ ⭑ |
| Srinivasamurthy, 2013 ^33^ | Retrospective | ⭑ ⭑ | ⭑ | ⭑ ⭑ ⭑ |
| Keskin, 2015 ^34^ | Retrospective | ⭑ ⭑ | ⭑ | ⭑ ⭑ |
| Arezzo, 2015 ^35^ | Retrospective | ⭑ ⭑ ⭑ | ⭑ | ⭑ ⭑ |
| Strangio, 2015 ^36^ | Prospective | ⭑ ⭑ ⭑ ⭑ | ⭑ | ⭑ ⭑ |
| Kuehn, 2016 ^37^ | Retrospective | ⭑ ⭑ | ⭑ | ⭑ ⭑ ⭑ |
| Mussetto, 2017 ^38^ | Retrospective | ⭑ ⭑ ⭑ | ⭑ | ⭑ ⭑ |
| Milito, 2017 ^39^ | Prospective | ⭑ ⭑ | ⭑ | ⭑ ⭑ |
| Mencio, 2018 ^40^ | Retrospective | ⭑ ⭑ | ⭑ | ⭑ |
| Jimenez-Rodriquez, 2018 ^41^ | Prospective | ⭑ ⭑ ⭑ | ⭑ ⭑ | ⭑ ⭑ ⭑ |
| Rottoli, 2018 ^42^ | Prospective | ⭑ ⭑ ⭑ | ⭑ | ⭑ ⭑ ⭑ |
| Katz, 2018 ^43^ | Retrospective | ⭑ ⭑ | ⭑ | ⭑ ⭑ ⭑ |
| Boschetti, 2018 ^44^ | Retrospective | ⭑ ⭑ | ⭑ | ⭑ ⭑ ⭑ |
| Huisman, 2019 ^45^ | Retrospective | ⭑ ⭑ ⭑ ⭑ | ⭑ | ⭑ ⭑ ⭑ |
| Kantowski, 2020 ^46^ | Retrospective | ⭑ ⭑ ⭑ | ⭑ ⭑ | ⭑ ⭑ ⭑ |
| Abdalla, 2020 ^47^ | Prospective | ⭑ ⭑ ⭑ ⭑ | ⭑ ⭑ | ⭑ ⭑ ⭑ |
| Wereen, 2020 ^48^ | Retrospective | ⭑ ⭑ ⭑ | ⭑ ⭑ | ⭑ ⭑ ⭑ |
| Kuhn, 2020 ^49^ | Prospective | ⭑ ⭑ ⭑ | ⭑ ⭑ | ⭑ ⭑ ⭑ |
| Jagielski, 2020 ^50^ | Prospective | ⭑ ⭑ ⭑ | ⭑ ⭑ | ⭑ ⭑ |
| Keshvari, 2020 ^51^ | Prospective | ⭑ ⭑ | ⭑ | ⭑ ⭑ |
