# Supplementary material for: Endoscopic vacuum therapy and early surgical closure after pelvic anastomotic leak: meta-analysis of bowel continuity rates
Source: Br J Surg. 2022 May 30;109(9):822–31. doi: 10.1093/bjs/znac158 (PMC10364759; doi:10.1093/bjs/znac158)
Supplement: znac158_Supplementary_Data [file znac158_supplementary_data.zip › Supplementary_Appendix_1.docx]

**Appendix S1:** *Search strategy and information resources*

With the aid of an expert librarian, a systematic review was performed in PubMed, MEDLINE and Cochrane Central Register of Controlled Trials databases, from database inception to November 2021 for published articles with relevant evidence regarding the EVT with or without ESC for colorectal AL. The combined terms used for the search were: ("Colorectal Surgery” OR "Colorectal Neoplasms" OR "Intestine, Large" OR "Rectal Diseases" OR "Colitis, Ulcerative" OR colo* OR rectal* OR rectum* OR anal OR ulcerative colitis OR colitis ulcer*) AND ("Anastomotic Leak" OR leak* OR insufficiency OR anastomo*) AND ("Negative-Pressure Wound Therapy" OR "Vacuum" OR vacuum OR EVAC OR transanal closure* OR trans-anal closure* OR early surgical closure OR endosponge* OR endo-sponge* OR negative pressure*).
