# Supplementary figures and images for: Endoscopic vacuum therapy and early surgical closure after pelvic anastomotic leak: meta-analysis of bowel continuity rates
Source: Br J Surg. 2022 May 30;109(9):822–31. doi: 10.1093/bjs/znac158 (PMC10364759; doi:10.1093/bjs/znac158)

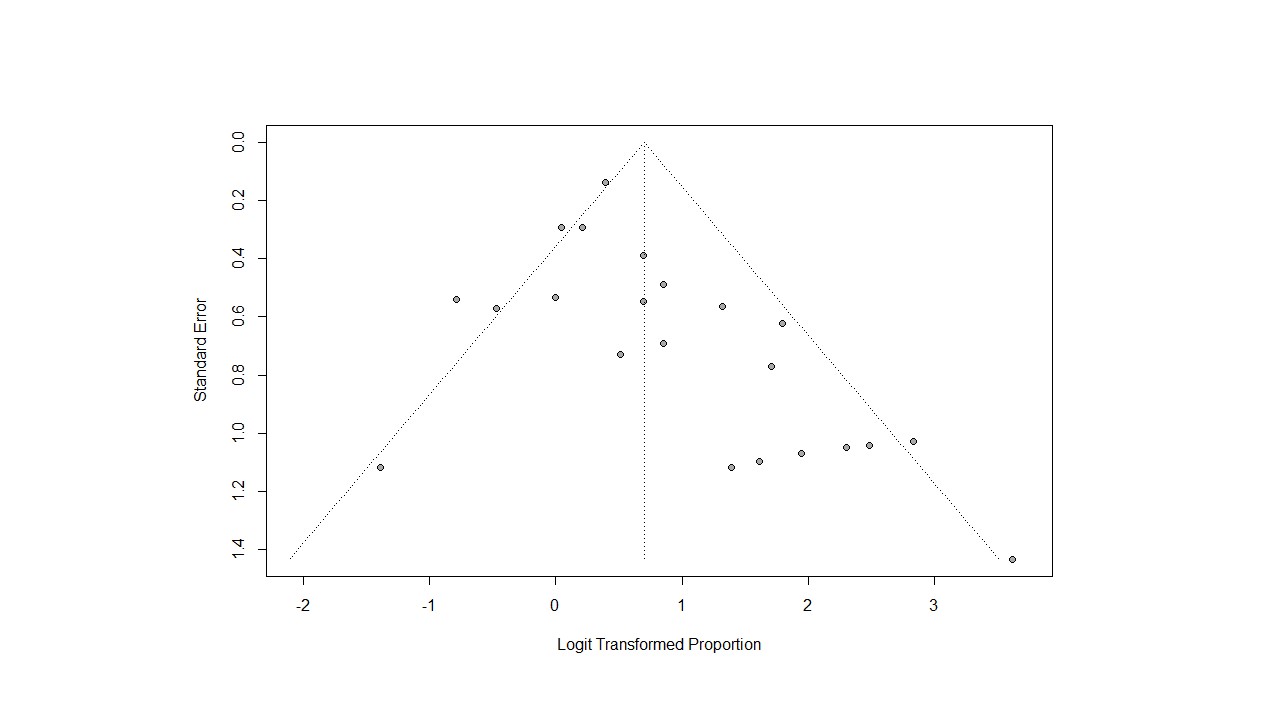

Supplement: znac158_Supplementary_Data [file znac158_supplementary_data.zip › Supplementary_Figure_1.jpg]
